# Genetic susceptibility loci for *Chlamydia trachomatis* endometrial infection influence expression of genes involved in T cell function, tryptophan metabolism and epithelial integrity

**DOI:** 10.3389/fimmu.2022.1001255

**Published:** 2022-09-29

**Authors:** Wujuan Zhong, Avinash Kollipara, Yutong Liu, Yuhan Wang, Catherine M. O’Connell, Taylor B. Poston, Kacy Yount, Harold C. Wiesenfeld, Sharon L. Hillier, Yun Li, Toni Darville, Xiaojing Zheng

**Affiliations:** ^1^ Department of Biostatistics, University of North Carolina at Chapel Hill, Chapel Hill, NC, United States; ^2^ Department of Pediatrics, University of North Carolina at Chapel Hill, Chapel Hill, NC, United States; ^3^ The University of Pittsburgh School of Medicine and the Magee-Womens Research Institute, Pittsburgh, PA, United States; ^4^ Department of Genetics, University of North Carolina at Chapel Hill, Chapel Hill, NC, United States

**Keywords:** *Chlamydial trachomatis*, upper genital tract infection in women, genetic marker for host susceptibility, cis-eQTLs, blood T cells and T cell subsets, CD151, integrated analysis of DNA genotypes and human blood transcriptome

## Abstract

**Objectives:**

Identify genetic loci of enhanced susceptibility to *Chlamydial trachomatis (Ct)* upper genital tract infection in women.

**Methods:**

We performed an integrated analysis of DNA genotypes and blood-derived mRNA profiles from 200 *Ct-*exposed women to identify expression quantitative trait loci (eQTL) and determine their association with endometrial chlamydial infection using a mediation test. We further evaluated the effect of a lead eQTL on the expression of *CD151* by immune cells from women with genotypes associated with low and high whole blood expression of *CD151*, respectively.

**Results:**

We identified *cis*-eQTLs modulating mRNA expression of 81 genes (eGenes) associated with altered risk of ascending infection. In women with endometrial infection, eGenes involved in proinflammatory signaling were upregulated. Downregulated eGenes included genes involved in T cell functions pivotal for chlamydial control. eGenes encoding molecules linked to metabolism of tryptophan, an essential chlamydial nutrient, and formation of epithelial tight junctions were also downregulated in women with endometrial infection. A lead eSNP rs10902226 was identified regulating *CD151*, a tetrospanin molecule important for immune cell adhesion and migration and T cell proliferation. Further *in vitro* experiments showed that women with a CC genotype at rs10902226 had reduced rates of endometrial infection with increased *CD151* expression in whole blood and T cells when compared to women with a GG genotype.

**Conclusions:**

We discovered genetic variants associated with altered risk for *Ct* ascension. A lead eSNP for *CD151* is a candidate genetic marker for enhanced CD4 T cell function and reduced susceptibility.

## Introduction

*Chlamydia trachomatis (Ct)* is the most commonly reported bacterial infection in the US. Infection is often asymptomatic and consequently untreated. No vaccine is available. In about 50% of women (1), infection can ascend from the cervix to the upper genital tract (uterus and fallopian tubes), potentially resulting in pelvic inflammatory disease (PID) and leading to devastating long-term sequela including chronic pelvic pain and infertility (2). In previous studies we have identified behavioral, biological, blood transcriptional, cervical cytokine, and *Ct* antigen-specific T cell and antibody responses, associated with altered risk of endometrial *Ct* infection (1, 3–6). However, a major research gap that remains is the lack of biomarker(s) that can identify women at elevated risk of disease. DNA variants, such as single nucleotide polymorphisms (SNPs) associated with enhanced risk of endometrial ascension could serve as biomarkers for susceptibility, promoting more effective screening that can be assessed independent of infection.

Several candidate gene and genome wide association studies (GWAS) of infertile women have reported genetic variants associated with altered risk of infertility, and used multiple parameters to link infertility with prior *Ct* exposure (7–11). However, development of infertility can be multifactorial, and confounded by occurrence of other sexually transmitted infections, behavioral, biological, and/or environmental factors. Although GWAS supports systematic detection of genetic variation when compared to candidate gene studies which are inherently biased, using GWAS to identify SNPs associated with ascending *Ct* infection would require thousands of women with defined endometrial *Ct* infection status. Furthermore, most loci identified by GWAS are not mapped to protein-coding regions, so the mechanism underlying their effect can be difficult to interpret.

An intermediate trait such as ascension of *Ct*, a prerequisite for tubal factor infertility, provides a substantial advantage to gene discovery studies. In this study, we analyzed blood samples from 200 *Ct*-exposed women with known endometrial *Ct* infection status with the goal of identifying expression quantitative trait loci (eQTLs) that modulate expression of genes associated with altered risk of *Ct* ascension. eQTLs are genomic SNPs (eSNPs) that influence expression of eGenes at the level of their regulatory elements. Our analysis focused on identifying SNPs in a *cis* window spanning a 1Mb region up- and downstream of the transcriptional start site of each gene. In our previous transcriptional profiling of women with symptomatic *Ct* PID and histologic endometritis, we determined that gene pathways upregulated in whole blood, e.g., type I interferon and myeloid cell activation, paralleled responses detected in endometrial tissue and cervical secretions, making blood eQTLs attractive candidate biomarkers predictive of risk for *Ct*-induced disease (3).

We found that *cis*-eQTLs altered the risk of ascending infection by modulating expression of 81 eGenes (FDR<0.2). eGenes involved in innate and adaptive immune responses, cellular trafficking, and metabolic pathways were associated with differential risk of endometrial *Ct*, suggesting that these eGenes and their regulating cis-eQTLs may serve as candidate genetic biomarkers of *Ct* ascension risk.

Of particular interest was a *cis*-eQTL modulating *CD151* expression that was significantly linked to risk of ascension. CD151 is a member of the tetraspanin family and has been associated with immune cell migration and adhesion and identifies T cells with hyperproliferative capabilities (12–15). Examination of genotypes for the lead *cis*-eQTL of *CD151* revealed women carrying a CC genotype displayed elevated whole blood and T cell expression of *CD151* with reduced risk of endometrial infection, when compared to women with a GG genotype. This CD151-associatead eQTL could serve as a potential biomarker of risk for endometrial *Ct* infection.

## Methods

This study complied with the Declaration of Helsinki guidelines and all study participants provided written informed consent prior to initiation of study procedures. The Institutional Review Boards for Human Subjects Research at the University of North Carolina, and the University of Pittsburgh approved the study.

### Study population

This study used whole blood collected from cis-gender female participants recruited into two cohorts: the Anaerobes and Clearance of Endometritis (ACE) cohort, comprised of women (age 16-40 years) who participated in a clinical trial (NCT01160640) comparing antibiotic regimens for the treatment of clinically diagnosed PID (16), and the T cell Response Against Chlamydia (TRAC) cohort, comprised of women with recognized risk factors for *Ct* who had cervicitis or were asymptomatic (age 15-35 years) (1). Participants in both cohorts were recruited at the University of Pittsburgh during 2011-2015. Clinical and microbiological data for the ACE and TRAC cohorts, mRNA transcriptional profiling, and DNA genotyping were generated as described previously (1, 3, 11, 16). DNA and mRNA extracted from whole blood samples from women with *Ct* infection in ACE and from all TRAC participants were used in this study. After quality control procedures, data from 200 women, including 57 uninfected, 71 cervical only infected (Endo-), and 72 with both cervical and endometrial infection (Endo+), were analyzed for eQTL mapping and mediation test for *Ct* ascension.

### Source and methodology for endometrial sampling

Endometrial sampling was obtained transcervically at enrollment after careful preparation of the cervix. The exocervix and endocervical canal were each cleansed with two applications of povidone alcohol. In order to prevent antiseptic solution interfering with microbiologic assays, we then dried the excess antiseptic in the endocervix with two sterile swabs. A sterile suction curette (Unimar Pipelle de Cornier, Cooper Surgical, Shelton, Connecticut) was placed into the endometrial cavity using sterile technique, and a tissue sample was aspirated into the cannula. After removal of the catheter, the tissue specimen was discharged into a sterile Petri dish. Tissue proximal to the sampling portal of the cannula was placed in 10% formalin fixative. In order to further minimize the contamination of the specimen from the cervix, a swab absorbed 5 mm of distal tissue for qualitative nucleic acid amplification testing (Aptima).

### Genotyping, imputation and data quality control

Genotyping was performed with the Illumina HumanOmniExpressExome-8 v1.2 BeadChip array. Markers with call rate < 95%, deviation from Hardy-Weinberg equilibrium (P < 1.00E−6), or more than two alleles were removed from subsequent imputation. Samples were also screened for relatedness based on identity by descent, and two samples with subject relatedness (PI_HAT > 0.185) were filtered [Anderson CA, 2010]. We phased and imputed post-QC genotype data using the 1,000 Genomes phase 1 dataset (17), SHAPEIT software (version 2.837) (18, 19), and BEAGLE software (version 4.1) (20). After imputation, variants with poor imputation quality (Beagle r2 < 0.1) and low minor-allele frequency (MAF < 0.1) were filtered.

### Gene expression microarray data acquisition and processing

Total RNA was isolated from blood and analyzed *via* microarray (Illumina Human HT12 v3.0 expression beadchip) as described previously (3). Expression profiles can be accessed from GEO (Gene Expression Omnibus) (https://www.ncbi.nlm.nih.gov/geo/query/acc.cgi?acc=GSE110106). Transcripts were quantile normalized (21) and log2 transformed. Batch effects were measured by guided principal component analysis (22), and corrected using ComBat (23).

### Population stratification and confounding factors

We used Plink (version 1.9) (24) (24)[Chang CC, 2015. www.cog-genomics.org/plink/1.9/] to control for population stratification. Population stratification was adjusted using the first 15 principal components, which explained >80% of the total variance. Oral contraceptive use, and gonorrhea co-infection were selected as covariates in the final model (1), and included in downstream analysis. We previously established that these covariates are risk factors for ascending infection by stepwise logistic regression of cross-sectional enrollment data, with the α to enter set at P ≤ 0.2 and the α to be maintained set at P ≤ 0.1.

### PEER factor analysis

We applied the probabilistic estimation of expression residuals (PEER) method (25), to infer and account for confounding factors affecting gene expression levels. We set maximum relevant factors in PEER to 50, then used the PEER-processed residuals of gene expression for downstream analyses. PEER factor analysis infers broad variance components in the measurements and is used to correct for observed and hidden confounding factors including bacterial burden (26–28). It has been demonstrated to correct blood cell compositions successfully in a similar, large blood-derived eQTL study (26).

### Heritability calculations

Heritability was calculated *via* a linear mixed model using GCTA (Genome-wide Complex Trait Analysis) software (29).

### *cis*-eQTL mapping

eQTLs were defined as *cis* if eSNPs were located within 1 Mb of the exon boundaries flanking a gene (eGene). We conducted single locus eQTL mapping using a linear regression model with adjustment of covariates and population stratification. Infection status (uninfected vs. infected), and genotype-infection interaction were included in the model to enable the detection of response eQTLs displaying varying effects between uninfected and infected women, and constant eQTLs with consistent effects regardless of infection status. Presence of genotype-infection interaction was examined with a cutoff p-value of 0.05. In the absence of significant genotype-infection interaction, a main effect model was used. Otherwise, a two degree of freedom test was performed. This compared a model in which transcript expression depends on the additive effects of a SNP and infection status together with their interaction term, to a model in which expression depends only on infection status. All analyses were conducted using R. We used all association p-values in the *cis* region to estimate the false-discovery rate (FDR-qvalue) using the qvalue package in R. LocusZoom (30) was used for the regional visualization of eQTL results on the basis of linkage disequilibrium (LD) ascertained from samples used in this study.

Associations of genotype at the lead cis-eQTL at the *CD151* locus and expression of *CD151* in uninfected participants and infected participants respectively, were tested using a linear regression model with adjustment of covariates and population stratification.

### Mediation test for *cis*-eQTL on ascending infection

We developed a generalized multi-SNP mediation intersection-union test to rigorously assess the effects of multiple eSNPs on ascension through their alteration of eGene expression (31). The locations and FDR of significant mediator eGenes were visualized using Circos software (32).

### Analyses of CD151 expression on T cells

We used extreme phenotype sampling to select samples for evaluation of the genetic effect of the lead SNP on *CD151* expression in blood T cells and T cell subsets after *Ct* stimulation using freshly thawed peripheral blood mononuclear cell (PBMCs). This is a well-established strategy for achieving statistical power to detect genotype-phenotype associations in the face of limited sample sizes with the rationale that the phenotypic extremes are enriched for either deleterious or protective variants (33, 34). For a SNP with an additive effect, the z-extreme sampling design, where extremes are defined based on “residual phenotype” after adjustment for non-genetic covariates, provides better power than random sampling (35). The phenotypic extremes in this study were defined as all women homozygous for the lead eSNP at the *CD151* locus with the upper or lower 20% extreme whole blood *CD151* expression after adjustment of covariates. We selected 6 women from each of two extreme groups by random sampling. After sampling, all 6 women from the upper extreme had CC genotype, and all 6 women from the lower extreme carried GG genotype.

Peripheral blood mononuclear cells (PBMCs) were thawed in T cell media (10% filtered, heat inactivated FBS, 5 mL of 1M HEPES, 5 mL of 200mM L-Glutamine, 5 mL of 100mM Sodium pyruvate, 200 μL of 100 mg/mL vancomycin and 25 mg of gentamicin) containing benzonase (50 units/mL of media; Sigma-Aldrich; Cat # E1014) followed by a wash in plain RPMI media and enumerated. For T cell evaluations, 1.5 x 106 cells were incubated in media alone in a 96 well plate, or wells were coated with 10 μg/mL of anti-CD3 (UCHT1) and 5 μg/mL of anti-CD28 (28.2) or pooled gamma-irradiated *Ct* EB/RBs from serovars D, F and H (5 μg/mL) and incubated for 7 days at 37°C in a 5% CO2 incubator with media replenishment every 24 hrs. Cells were harvested and stained with 1:100 of Zombie UV fixable viable dye (BioLegend, Cat # 423107) in 1x PBS for 15 min at room temperature. Cells were subsequently washed with cell staining buffer (BioLegend, Cat # 420201) before staining with an optimized concentration of antibody cocktail comprising CD45-BV510 (BioLegend, Clone: HI30, Cat # 304036), CD3-APC (BioLegend, Clone: HIT3a, Cat # 300312), CD4-PerCP-Cy5.5 (BioLegend, Clone: RPA-T4, Cat # 300530), CD8-AF700 (BioLegend, Clone: SK1, Cat # 344724), CD45RA-BV421 (BioLegend, Clone: HI100, Cat # 304130), CCR7-BV605 (BioLegend, Clone: G043H7, Cat # 353224), CD151-PE (BD Biosciences, Clone: 14A2.H1, Cat # 556057) and Ki67-PE/Cy7 (BD Biosciences, Clone: B56, Cat # 561283) for 30 min at 4°C in dark. After staining, cells were washed twice and fixed with 2% PFA. Fixed cells were washed and fluorescence acquired on a BD LSR II, 7 Laser cytometer. Wilcoxon rank-sum test was used to compare percentages of CD151+ T cells and CD151 MFI between two genotype groups (GG vs. CC); paired samples Wilcoxon test was used to compare groups before and after *in-vitro* stimulation.

## Results

### Participant characteristics

The characteristics of participants in this study are summarized in **Table 1**. Only oral contraceptive pill use and co-infection with *Neisseria gonorrhoeae* were significantly different among the three groups with rates being higher in women with endometrial infection, consistent with our previous report (1). Consequently, these factors were adjusted as covariates in the models used in this study.

**Table 1:** Population characteristics.

Characteristics	Number (%)^a^				P value
	Overall	Endo+	Endo-	Uninfected	
Total number of women per group	200	72	71	57	
Age, y, median (range)	21 (18 - 35)	20 (18 - 28)	21 (18 - 35)	21 (18 - 34)	0.162
Race/Ethnicity					
African American	130 (65)	44 (61.11)	51 (71.83)	35 (61.4)	0.415
White	38 (19)	17 (23.61)	10 (14.08)	11 (19.3)	
Hispanic or Latino	12 (6)	1 (1.39)	5 (7.04)	6 (10.53)	
Multiracial	24 (12)	8 (11.11)	8 (11.27)	8 (14.04)	
Other	7 (3.5)	2 (2.78)	2 (2.82)	3 (5.26)	
Marital status					0.329
Single	180 (90)	64 (88.89)	66 (92.96)	50 (87.72)	
Living with partner	16 (8)	5 (6.94)	4 (5.63)	7 (12.28)	
Divorced or separated	2 (1)	2 (2.78)	0 (0)	0 (0)	
Widowed	1 (0.5)	0 (0)	1 (1.41)	0 (0)	
Education					0.169
Less than high school graduate	29 (14.5)	9 (12.5)	13 (18.31)	7 (12.28)	
High school graduate or GED degree	76 (38)	25 (34.72)	33 (46.48)	18 (31.58)	
Some college work	65 (32.5)	25 (34.72)	15 (21.13)	25 (43.86)	
College graduate	12 (6)	5 (6.94)	3 (4.23)	4 (7.02)	
Post-grad work	1 (0.5)	0 (0)	0 (0)	1 (1.75)	
Vocational training	16 (8)	7 (9.72)	7 (9.86)	2 (3.51)	
Insurance					0.084
Private	43 (21.5)	23 (31.94)	10 (14.29)	12 (21.05)	
Medicaid	102 (51)	29 (40.28)	43 (61.43)	28 (49.12)	
Other insurance	12 (6)	4 (5.56)	6 (8.57)	2 (3.51)	
None	41 (20.5)	15 (20.83)	11 (15.71)	15 (26.32)	
Smoking	105 (52.5)	34 (47.22)	38 (53.52)	33 (57.89)	0.531
Marijuana use	73 (36.5)	27 (37.5)	26 (36.62)	20 (35.09)	0.975
Alcohol use	100 (50)	38 (52.78)	33 (46.48)	29 (50.88)	0.706
Birth control					
OCPs	24 (12)	13 (18.06)	4 (5.63)	4 (7.02)	0.038
DMPA	26 (13)	7 (9.72)	14 (19.72)	5 (8.77)	0.148
Intrauterine device	25 (12.5)	8 (11.11)	7 (9.86)	10 (17.54)	0.456
Condoms	112 (56)	37 (51.39)	42 (59.15)	33 (57.89)	0.725
Abstinence	17 (8.5)	8 (11.11)	6 (8.45)	3 (5.26)	0.513
Coitus interruptus	63 (31.5)	22 (30.56)	25 (35.21)	16 (28.07)	0.682
Infection status at enrollment					
co-infection of neisseria gonorrhoeae	23 (11.5)	18 (25)	10(14.09)	0 (0)	2.96E-05
co-infection of mycoplasma genitalium	34 (17)	17 (23.61)	22 (30.98)	11 (19.30)	0.317
co-infection of Trichomonas vaginalis	35 (17.5)	8 (11.11)	16 (22.54)	11 (19.3)	0.168
Bacterial vaginosis at enrollment					0.984
Negative bacterial vaginosis (Nugent Score=0~3)	51 (25.5)	20 (27.78)	18 (25.35)	13 (22.81)	
Intermediate bacterial vaginosis (Nugent Score=4~6)	33 (16.5)	12 (16.67)	12 (16.9)	9 (15.79)	
Positive bacterial vaginosis (Nugent Score=7~10)	115 (57.5)	40 (55.56)	41 (57.75)	34 (59.65)	

^a^Data are number (%) of subjects, unless otherwise indicated.

GED, general education development; OCP, oral contraceptive pills; DMPA, depot medroxyprogesterone; Endo+, both endometrial and cervical infection; Endo-, cervical only infection.

### Heritability and *cis*-eQTLs

We analyzed genotype data of ∼2.6 million SNPs and the expression profiles of 47,109 probe sets corresponding to 34,540 unique genes. The estimated mean narrow-sense heritability, h2, for variance of gene expression was 0.23 (standard error = 0.098, *P* = 0.009), suggesting significant genetic effects on gene expression. We identified 105,873 SNPs with effects in cis (<1 Mb) for 6,837 genes (FDR < 0.1, P < 2.17E-4). **Table 2** summarizes the identified cis-eQTLs.

**Table 2:** Discovery of cis-eQTLs^a^ (FDR<0.1) in blood from *Chlamydia trachomatis-*exposed women.


Number of variant-probeset pairs	143,779
Number of variant-gene pairs	131,654
Number of associated probesets	7,786
Number of associated genes	6,837
Number of associated Variants	105,873

^a^cis-eQTL with association p values < 2.17E-04, corresponding to FDR 0.1

### Genes mediating cis effects on ascending infection

**Figure 1:**
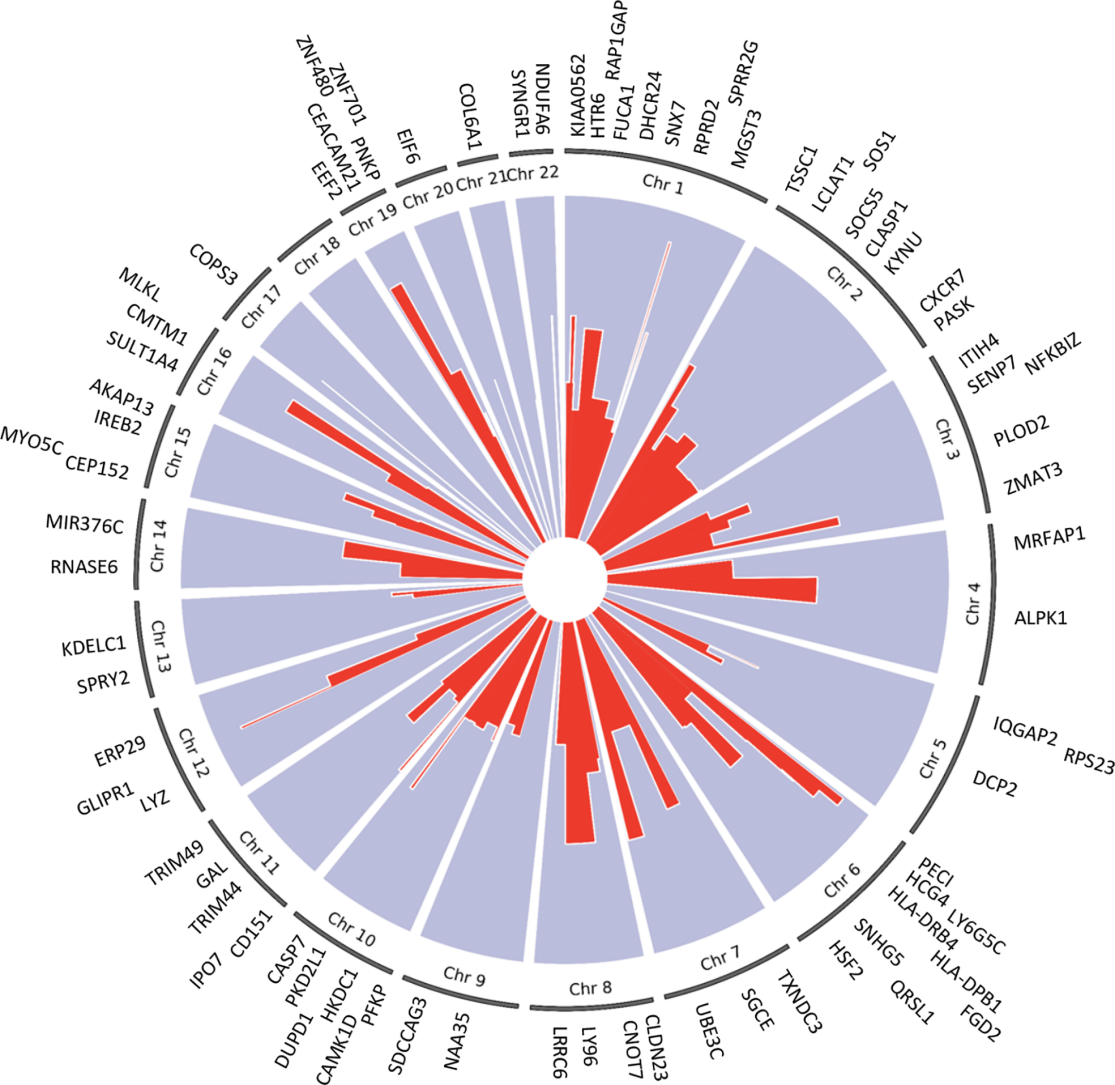
Circos plot of 81 eGenes mediating effects of cis-eQTLs on ascension (FDR <0.2) and their chromosome number. The -log 10(FDR) mediation test results for each eGene are represented by the red lines. The width of the red lines indicates the size of each gene. The eGenes are labeled at their respective chromosome base pair location boundaries.

**Figure 2:**
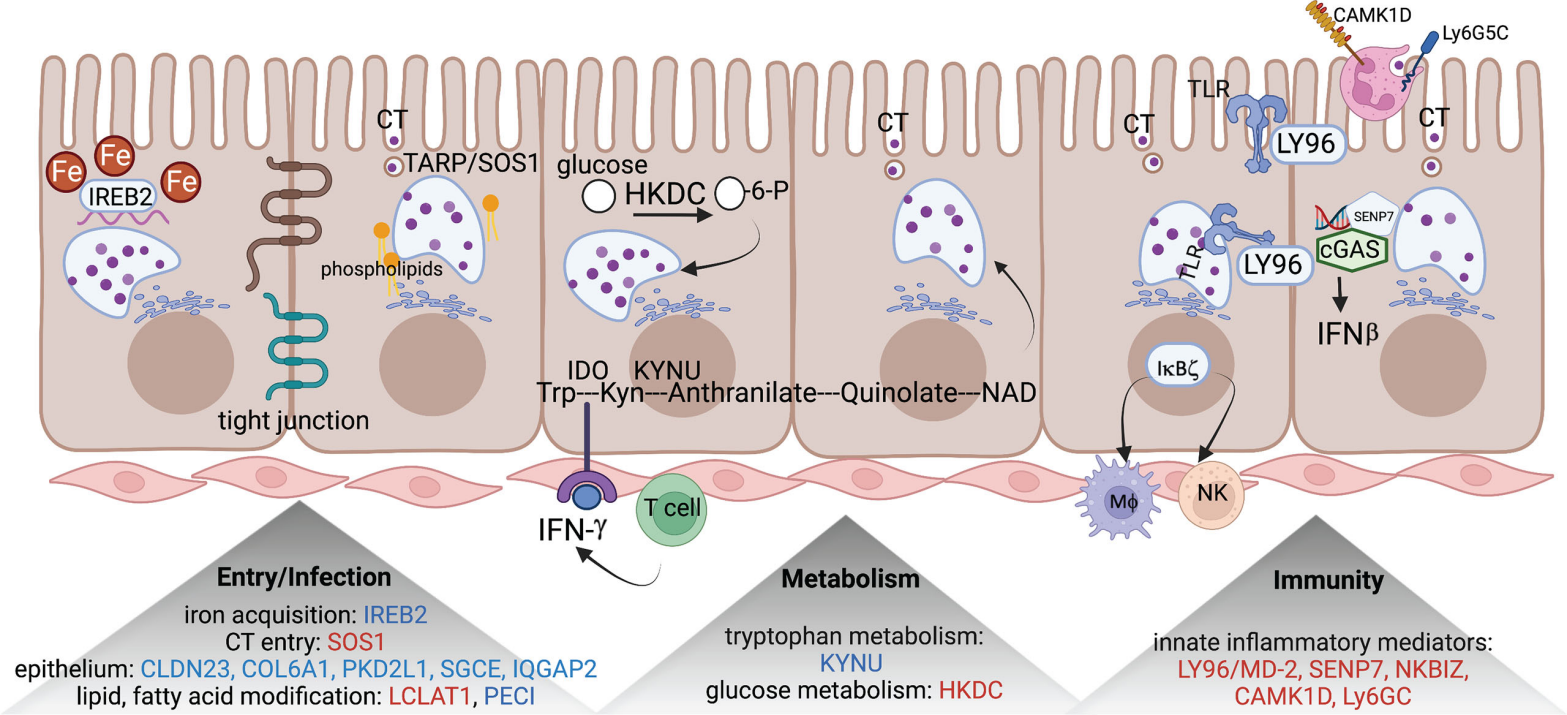
eGenes encoding proteins linked to biologic pathways involved in chlamydial infection. eGenes downregulated in women with endometrial infection are listed in blue and include: IREB2, an iron acquisition protein; multiple molecules involved in formation of tight junctions and epithelial integrity (CLDN23, COL6A1, PKD2L1, SGCE, and IQGAP2); PECI, that modifies fatty acids; and KYNU, important in tryptophan metabolism. eGenes upregulated in women with endometrial infection are listed in red and include SOS1, that encodes a protein that directly interacts with chlamydial TARP to facilitate entry; LCAT1 important for lipid modification; HKDC, glucose metabolism; and genes for multiple innate inflammatory mediators (LY96/MD-2, SENP7, NKBIZ, CAMK1D, and Ly6GC). The figure was created using BioRender.com.

**Figure 3:**
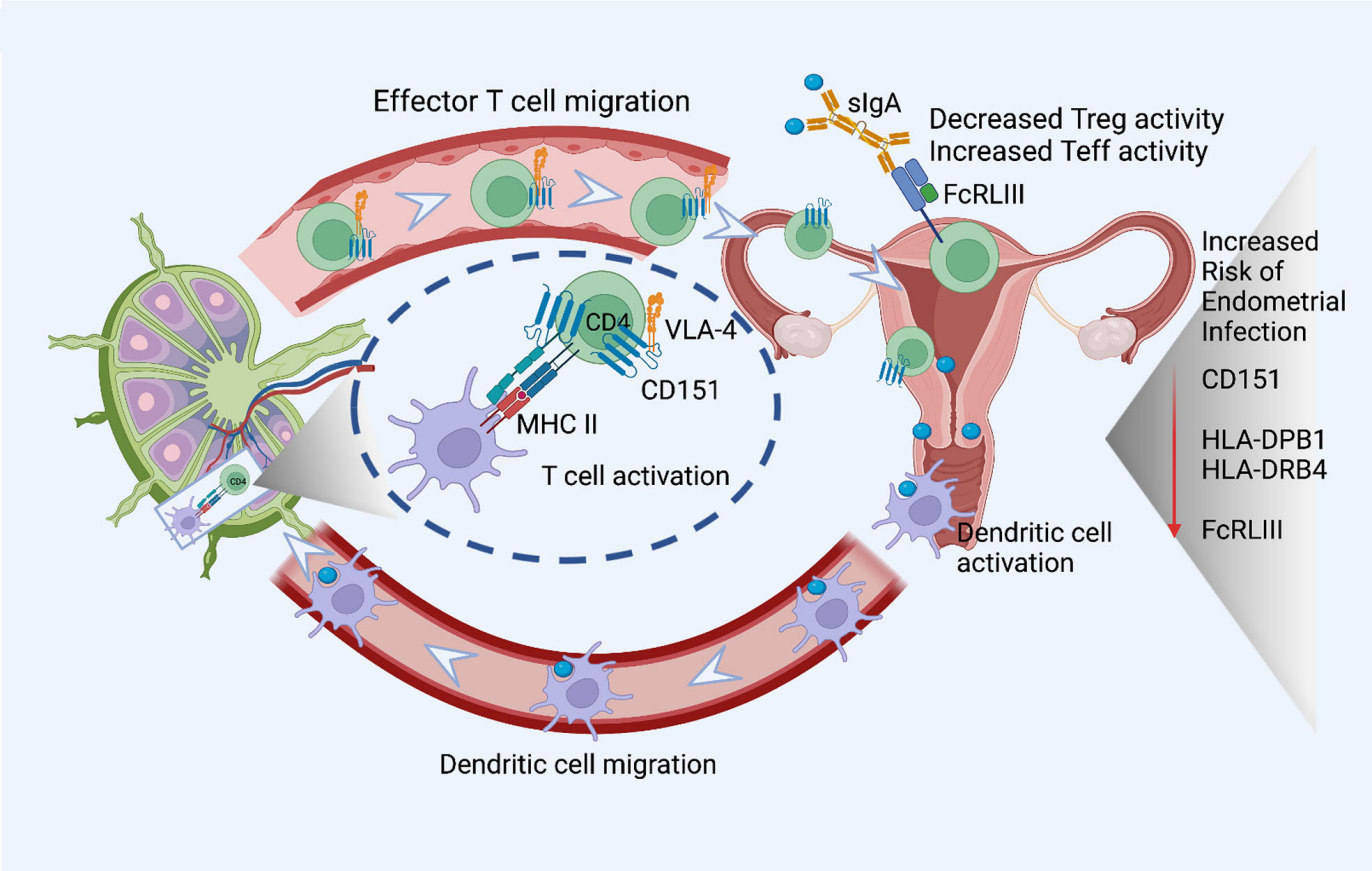
eGenes important for T cell activation, function, and migration downregulated in women with endometrial infection include: CD151, a tetraspanin intimately involved in the T cell/antigen presenting cell synapse that also interacts with integrins to promote T cell migration; MHC Class II molecules important for antigen presentation to CD4 T cells, HLA-DPB1 and HLA-DRB4; and FcRLIII, that binds bacterial-specific sIgA at mucosal surfaces to inhibit T suppressor functions and enhance T effector functions. The figure was created using BioRender.com.

We applied the generalized multi-SNP mediation intersection-union test (31) to the identified *cis*-eQTLs with FDR < 0.1, and determined that expression of 33 eGenes was increased in women with endometrial infection (FDR < 0.2, **Table 3**), and expression of 48 eGenes was decreased in women with endometrial infection (FDR < 0.2, **Table 4**). Distribution and size of these 81 eGenes, their positions within the chromosomes and -log 10(FDR) of mediation tests were demonstrated by circos plot (**Figure 1**). We have highlighted eGenes below that are related to biologic pathways involved in *Ct* infection and inflammation (**Figure 2**) and/or T cell mediated control/clearance (**Figure 3**).

**Table 3:** eGenes with increased expression in women with endometrial infection, and their corresponding cis-eQTLs and chromosomal locations (mediation test FDR^a^<0.2).

eGene symbol	Probe id^b^	Full gene name	Lead eQTL^c^	Chr
ALPK1	540390	Alpha-kinase 1	rs28688349	4
CAMK1D	4780452	Calcium/calmodulin-dependent protein kinase	rs61833192	10
CEACAM21	1190307	Carcinoembryonic antigen-related cell adhesion molecule21	rs3745936	19
CLASP1	5290215	Cytoplasmic linker associated protein 1	rs11122869	2
CMTM1	7510097	Chemokine-like factor (CKLF)-like MARVEL transmembrane domain	rs13331838	16
COPS3	1030551	COP9 signalosome complex subunit 3	rs4985752	17
CXCR7	450424	C-X-C chemokine receptor type 7	rs7603272	2
DHCR24	4480341	24-Dehydrocholesterol Reductase	rs3170766	1
DUPD1	7040025	Dual specificity phosphatase 29	rs9415136	10
HKDC1	870301	hexokinase domain containing 1	rs9645499	10
HSF2	130121	Heat shock factor 2	rs578868	6
IPO7	5720538	Importin 7	rs10840234	11
LCLAT1	1190228	Lysocardiolipin acyltransferase	rs829669	2
LRRC6	2350477	Leucine-rich repeat containing protein 6	rs1834439	8
LY6G5C	3520647	Leukocyte antigen-6	rs409558	6
LY96	70167	Lymphocyte antigen 96, also myeloid differentiation factor 2 (MD-2)	rs11775465	8
NAA35	2470746	N-alpha-acetyltransferase 35	rs10117897	9
NDUFA6	6580132	NADH : Ubiquinone Oxidoreductase Subunit A6	rs6002607	22
NFKBIZ	2470348	Nuclear factor IkBz	rs2929922	3
PASK	4150100	Serine/threonine-protein kinase	rs2240538	2
PFKP	2360452	Platelet isoform of phosphofructokinase	rs4881110	10
PLOD2	460338	Procollagen-lysine,2-oxoglutarate 5-dioxygenase 2	rs13073669	3
PNKP	7610184	Polynucleotide kinase phosphatase	rs3810268	19
SDCCAG3	60706	Serologically defined colon cancer antigen-3	rs3812550	9
SENP7	2350468	Sentrin-specific protease 1	rs13322987	3
SNHG5	1050475	small nucleolar RNA host gene 5	rs9444348	6
SOCS5	6370379	Suppressor of cytokine signaling 5	rs1109380	2
SOS1	2140519	Son of sevenless 1, guanine nucleotide exchange factor	rs13031871	2
SPRY2	6590575	Sprouty receptor tyrosine kinase signaling antagonist	rs2478196	13
SYNGR1	6770768	Synaptogyrin-1	rs909685	22
TSSC1	5700722	Tumor-Suppressing Subchromosomal Transferable complex	rs9678647	2
ZNF480	3520537	Zinc finger protein 480	rs8112051	19
ZNF701	3780280	Zinc finger protein 701	rs35429660	19

^a^FDR: False discovery rate controlled by Benjamini-Hochberg procedure for mediation test.

^b^Probe ids for corresponding genes in Illumina Human HT12 v3.0 expression beadchip.

^c^Lead eQTL: the most significant eQTL for the target gene.

**Table 4:** eGenes with decreased expression in women with endometrial infection, and their corresponding cis-eQTLs and chromosomal locations (mediation test FDR^a^<0.2).

eGene symbol	Probe id^b^	Full gene name	Lead eQTL^c^	Chr
AKAP13	650050	A-kinase anchor protein-13	rs8037947	15
CASP7	7650324	Caspase 7	rs60253032	10
CD151	6550259	Cell determinant 151	rs10902226	11
CD151	1940132	Cell determinant 151	rs7126859	11
CEP152	990189	Centrosomal Protein 152	rs73402203	15
CLDN23	1850156	Claudin-23	rs2409095	8
CNOT7	3120176	CCR4-NOT transcription complex 7	rs7386410	8
COL6A1	7570598	Type VI collagen	rs2330353	21
DCP2	6960349	Decapping Protein	rs66612320	5
EEF2	1580292	Eukaryotic Elongation Factor-2 Kinase	rs350835	19
EIF6	1850681	Eukaryotic initiation factor 6	rs2425043	20
ERP29	4120333	Endoplasmic reticulum stress protein	rs60258199	12
FCRL3	620167	Fc receptor-like 3	rs7528684	1
FGD2	7210717	RhoGEF and PH domain-containing protein 2	rs708015	6
FUCA1	2060121	alpha-L-fucosidase	rs12736774	1
GAL	60452	Galanin	rs11228490	11
GLIPR1	6220746	Glioma pathogenesis-related protein 1	rs10879911	12
HCG4	2690390	HLA Complex Group 4	rs116306799	6
HLA-DPB1	1050360	Human leukocyte antigen DPB1	rs116458532	6
HLA-DRB4	510079	Human leukocyte antigen DRB4	rs9272477	6
HTR6	4040465	5-hydroxytryptamine receptor 6	rs7550916	1
IQGAP2	4220440	IQ Motif Containing GTPase Activating Protein 2	rs56189196	5
IREB2	6840240	Iron-responsive element-binding protein 2	rs11637656	15
ITIH4	3710343	Inter-Alpha-Trypsin Inhibitor Heavy Chain 4	rs2071041	3
KDELC1	160209	Lys-Asp-Glu-Leu containing 1	rs2567774	13
KIAA0562	6650288	centrosomal protein 104kDa	rs7535775	1
KYNU	3460685	Kynureninase	rs352930	2
KYNU	7040142	Kynureninase	rs351685	2
LYZ	1690056	Lysozyme	rs7959452	12
MGST3	7160400	Microsomal Glutathione S-Transferase 3	rs2018265	1
MIR376C	6620288	MicroRNA 376c	rs4900470	14
MLKL	1450414	Mixed-lineage kinase domain-like protein	rs8060923	16
MRFAP1	2340372	Mortality factor on chromosome 4 related protein	rs4689022	4
MYO5C	1980246	Myosin5C	rs979259	15
PECI	6200133	Peroxisomal 3,2-trans-enoyl-CoA isomerase	rs6929344	6
PKD2L1	2760463	Polycystic kidney disease 2-like 1 protein; transient receptor potential polycystic 2 (TRPP2)	rs11593471	10
QRSL1	3190681	Glutaminyl-TRNA Amidotransferase Subunit -1	rs11968502	6
RAP1GAP	4890181	Ras-like small GTPase	rs7515855	1
RNASE6	130681	Ribonuclease A family member k6	rs8003813	14
RPRD2	3420377	Regulation of Nuclear Pre-mRNA Domain 2	rs4970971	1
RPS23	380070	40S ribosomal protein S23	rs2406911	5
SGCE	5290348	Sarcoglycan epsilon	rs12530498	7
SNX7	3850593	Sorting nexin -7	rs4908332	1
SPRR2G	1470630	Small Proline Rich Protein 2G	rs1998845	1
SULT1A4	5490594	Sulfotransferase family 1A4	rs62034325	16
TRIM44	1300239	Tripartite motif 44	rs2938188	11
TRIM49	450068	Tripartite motif 49	rs56004718	11
TXNDC3	6480403	Thioredoxin domain-containing protein 3	rs2717931	7
UBE3C	1690709	Ubiquitin Protein Ligase E3C	rs2366004	7
ZMAT3	3140543	Zinc finger matrin-type protein 3	rs9882786	3

^a^FDR: False discovery rate controlled by Benjamini-Hochberg procedure for mediation test.

^b^Probe ids for corresponding genes in Illumina Human HT12 v3.0 expression beadchip.

^c^Lead eQTL: the most significant eQTL for the target gene.

*SOS1*, encoding a nucleotide exchange factor that interacts with chlamydial translocated actin recruiting phosphoprotein (TARP) to facilitate chlamydial entry (36), was upregulated in women with endometrial infection, while several eGenes involved in formation of tight junctions and epithelial cell adhesion (*CLDN23* (37), *COL6A1* (38)(REFs), *PKD2L1* (39), *SGCE* (40), *IQGAP2* (41, 42)) were downregulated in women with endometrial infection (**Table 4**). Increased *Ct* entry into epithelial cells and compromised epithelial integrity are plausible mechanisms for increased risk of ascension.

eGenes also mapped to loci involved in phospholipid modification and fatty acid metabolism. *LCLAT1* (Lysocardiolipin Acyltransferase 1), exhibiting increased expression in women with endometrial infection (**Table 3**), encodes a protein that catalyzes the reacylation of lyso-cardiolipin to cardiolipin (43), a key step in cardiolipin remodeling, while PECI (**Table 4**), decreased in women with endometrial infection, encodes peroxisomal 3,2-trans-enoyl-CoA isomerase which catalyzes an isomerization step leading to beta-oxidation of unsaturated fatty acids (44). Since the chlamydial genome carries all the genes necessary for phospholipid and fatty acid synthesis and biochemical studies have confirmed that bacterial membrane biogenesis is achieved with autonomously synthesized phospholipids (45–47), it seems unlikely that these genes could influence chlamydial replicative success directly. However, host membrane trafficking pathways are hijacked to assemble the expanding inclusion membrane required to accommodate multiplying chlamydial progeny (48). Inhibition of host lipid synthesis/trafficking decreases the yield of *Ct* (49), and lipid biosynthesis inhibitors lead to premature rupture of the inclusion membrane (50), arguing that these eGenes may be important for maximizing infectious yield. An eGene involved in the regulation of iron homeostasis was also mapped. Down-regulated expression of the gene for iron-responsive element-binding protein 2 (IREB2) (**Table 4**), which regulates cellular iron homeostasis, might restrict intracellular growth of chlamydiae directly because iron restriction limits chlamydial infection (51) or indirectly through co-regulatory effects modulating tryptophan metabolism (52).

A metabolic pathway eGene with potential to impact *Ct* replication and development, *KYNU* which encodes kynureninase, was downregulated in women with endometrial infection (**Table 4**). Interferon-gamma released from immune cells infiltrating *Ct* infected tissues activates indoleamine 2,3-dioxygenase (IDO) which catalyzes the breakdown of tryptophan to kynurenine (KYN) (53). *Ct* lack several enzymes of the tryptophan biosynthetic pathway (54) and are largely dependent on cellular metabolism to access sufficient tryptophan to support replication (55). Kynureninase cleaves KYN to anthranilic acid, which eventually results in production of nicotinamide adenine dinucleotide (NAD). Since dioxygenases are partially regulated by end product inhibition (56), reduced KYN activity could increase tryptophan metabolism and reduce its availability to *Ct*. Fisher and colleagues determined that the chlamydial ATP/ADP translocase, Npt1, preferentially transports NAD, indicating that the bacteria scavenge this energy substrate from their host, despite their ability to complete *de novo* synthesis using folate (57). Finally, kynurenine metabolites are potent regulators of immune function (58). Thus, altered expression of *KYNU* may affect chlamydial growth through changes in the availability of tryptophan or NAD or immune response alterations. Another metabolic gene, encoding HKDC (hexokinase domain containing 1), that catalyzes ATP-dependent phosphorylation of glucose to G6P (59), a primary carbohydrate substrate for *Ct* (60, 61), was elevated in women with endometrial infection (**Table 3**).

Myeloid cell and innate inflammatory responses are primarily associated with development of reproductive tract tissue pathology during *Ct* infection rather than infection resolution (62, 63). *LY96*, encoding lymphocyte antigen 96, also called MD-2, which associates with TLR2 and TLR4 to increase their responsiveness to lipoproteins and LPS (64, 65), and chlamydial heat shock protein 60 (66), was upregulated in women with endometrial infection (**Table 3**). Other inflammatory genes that were upregulated in women with endometrial infection included *SENP7*, *CAMK1D*, and *Ly6G5C*. The protease encoded by *SENP7* potentiates the DNA sensor cyclic GMP-AMP synthase (cGAS) by relieving small ubiquitin-like-related modifier (SUMO) inhibition of cytosolic DNA sensing (67). cGAS is required for IFN-β expression during chlamydial infection in multiple cell types (68), and murine (69) and human studies (3) indicate IFN-β compromises resolution of chlamydial infection and exacerbates pathology. The calcium/calmodulin-dependent protein kinase, encoded by *CAMK1D*, regulates activation of neutrophils (70), and the protein encoded by *LY6G5C* regulates neutrophil migration (71).

A robust CD4 T cell response is key to elimination of *Ct* infection (72, 73). Women with endometrial infection exhibited downregulation of *CD151*, which encodes a protein that augments T cell activation, adhesion and migration (12, 13, 74–76). The eGene for FCRL3, which enhances generation of CD4 effector memory T cells at mucosal sites (77), and eGenes for HLA-DPB1, HLA-DRB4, MHC Class II molecules important for activation of CD4 T cells (78, 79) were also down-regulated (**Figure 3**). Previous work demonstrated that FCRL3 engagement inhibited regulatory T cell suppressive functions, and induced IL-17, IL-26, and IFNγ production, suggesting that FCRL3 engagement mediates a transition of regulatory T cells to a pro-inflammatory Th17-like phenotype. Secretory IgA (SIgA) is a FCRL3-specific ligand, suggesting pathogen-specific sIgA might drive mucosal regulatory T cell plasticity to help control infection (77). The associated decrease in expression of eGene for HLA-DPB1 and HLA-DRB4 could also diminish Class II presentation of chlamydial antigens that would further inhibit protective CD4 T cell responses. Increased expression of the eGene for SOCS5 in women with endometrial infection (**Table 3**) may reflect inhibition of STAT1 and STAT3 signaling necessary for the differentiation of Th1 and Th17 cells, respectively (80).

### *cis*-eQTLs at *CD151* and *HLA-DPB1* loci

**Figure 4:**
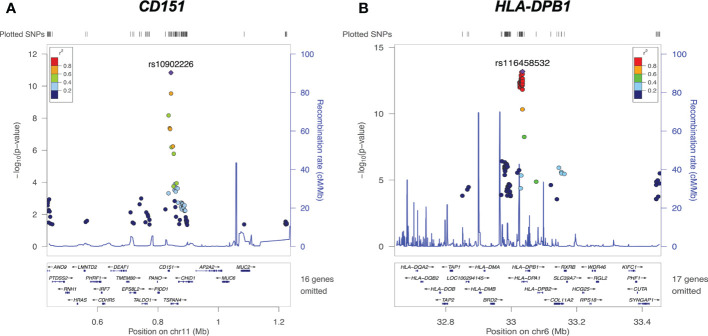
cis-eQTLs of *CD151 and HLA-DPB1.* **(A)** Regional plot on chromosome 11 *CD151* locus reveals the lead cis-eQTL rs10902226 (purple diamond) is significantly associated with *CD151* expression (P<10E-11). **(B)** Regional plot on chromosome 6 *HLA-DPB1* locus reveals the lead cis-eQTL rs116458532 (purple diamond) is significantly associated with *HLA-DPB1* expression (P<1.00E-13). For both graphs, each dot represents one SNP. X axis shows the physical location of the SNPs, gene names and flanking region; Y axis indicates the −log10 value (P-value) of the respective SNP. The color for each dot represents the pairwise linkage disequilibrium r2-value against respective lead cis-eQTL.

Examples of significant *cis*-eQTL signals at the *CD151* and *HLA-DPB1* loci are demonstrated in **Figure 4**. The lead eSNP (strongest eQTL) rs10902226 (**Figure 4A**) is located 4266 base pairs downstream of *CD151* gene and is a *TSPAN4* intronic variant. The lead eSNP rs10902226 (**Figure 4B**) is located 9363 base pairs upstream of *HLA-DPB1* gene and is a *HLA-DPA1* intronic variant. These two lead eSNPs are highly significantly associated with expression of *CD151* and *HLA-DPB1* with P<1.00E-11 and P<1.00E-13, respectively. Multiple nearby SNPs in LD (r2>0.6) with the lead eSNPs are also significant *cis*-eQTLs (P<1.00E-8 for *CD151* and P<1.00E-10 for *HLA-DPB1*, respectively), implying that both lead eSNPs are true.

**Figure 5:**
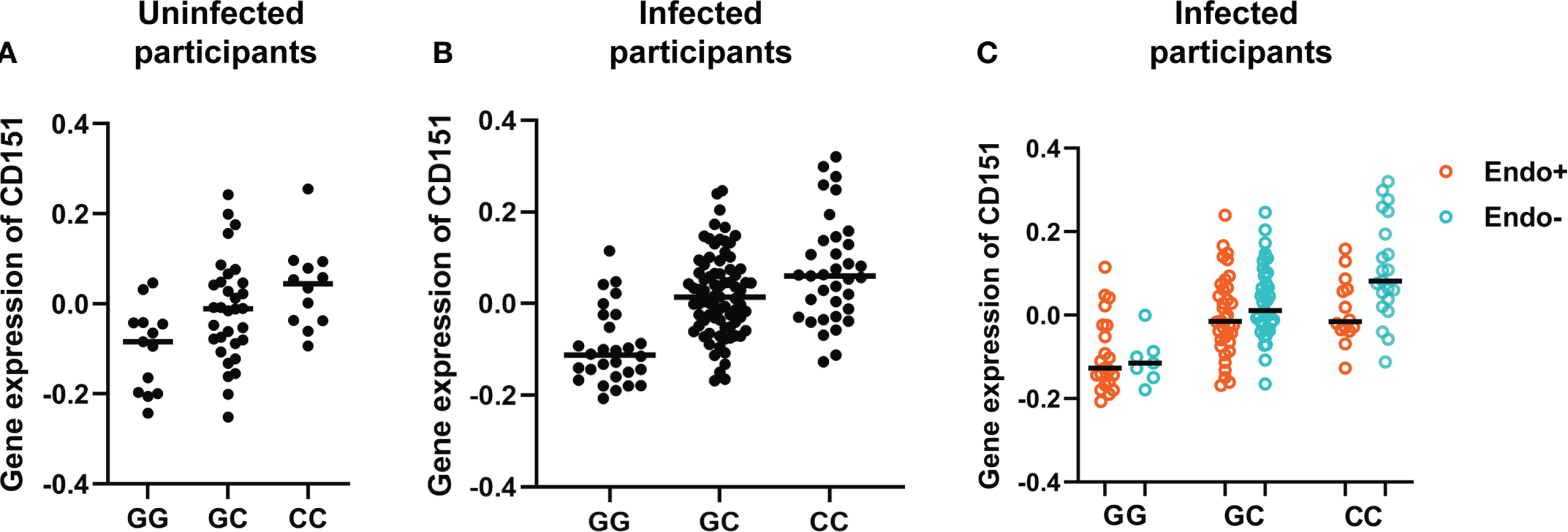
Genotype at the lead cis-eQTL rs10902226 at the *CD151* locus is associated with altered expression of *CD151* and risk of ascension. Using linear regression, in uninfected participants **(A)**, the SNP shows suggestive evidence to be an eQTL (P value = 0.0018), but it is not significant after multiple testing correction. In infected participants **(B)**, the SNP is a strong eQTL (P value = 6.7E-9) with an additive effect. In infected participants **(C)**, the effect of the C allele on CD151 expression and its relationship to endometrial infection can be visualized (P=0.012 by mediation test on this single SNP). This graph demonstrates that CC genotype is associated with increased CD151 expression and reduced occurrence of endometrial infection. In contrast, GG genotype is associated with lower CD151 expression and increased occurrence of endometrial infection. In all graphs, each dot represents one subject. The black line indicates the median expression of *CD151* in each genotype group.

The effect of genotypes on CD151 expression in uninfected and infected participants are further represented by **Figure 5**. **Figure 5A** presents the association of genotype at the lead cis-eQTL rs10902226 at the CD151 locus and expression of CD151 in uninfected participants and **Figure 5B** for infected participants. In uninfected participants (A), the SNP shows suggestive evidence to be an eQTL (P value = 0.0018), but it is not significant after multiple testing correction. In infected participants (B), the SNP is a strong eQTL (P value = 6.7E-9) with an additive effect. In (C) the effect of the C allele on CD151 expression and its relationship to endometrial infection can be visualized (P=0.012 by mediation test on this single SNP). This graph demonstrates that CC genotype is associated with increased CD151 expression and reduced occurrence of endometrial infection, in contrast GG genotype is associated with lower CD151 expression and increased occurrence of endometrial infection.

### T cell CD151 expression in women with CC and GG genotypes

CD151 is a member of the tetraspanin family (TM4SF) associated with immune cell migration and adhesion. It associates with multiple integrins and plays a role in regulating integrin trafficking and/or function (81). CD151 actively changes cell cycle control and cell death processes and identifies T cells with hyperproliferative capabilities (13).

**Figure 6:**
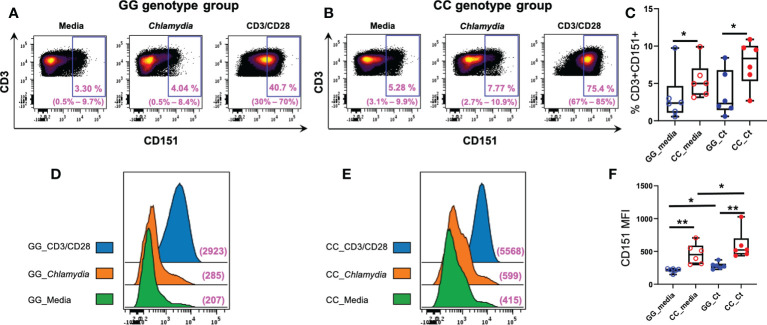
Association of *CD151* genotypes with CD3+ lymphocyte CD151 expression in *Ct* infected women. Each dot plot is a concatenated FCS file consisting of CD3+ cells from six women of **(A)** GG and **(B)** CC genotypes, respectively. Range of six individual patients within each group’s condition is in parentheses. **(C)** Frequencies of CD151+CD3+ cells in GG (n = 6; blue circles) and CC genotype (n = 6; red circles) women with (filled circles) and without (empty circles) *Ct* stimulation for 7 days. The boundaries of each box indicate the 25th and 75th percentiles; the line within each box indicates the median; whiskers indicate the 0th and 100th percentiles. Each dot represents one subject. The relative frequencies of CD3+CD151+ lymphocytes were significantly increased in the CC group with and without *Ct* stimulation compared to GG group by Wilcoxon Rank Sum Test. **(D)** Mean florescence intensities (MFI) of CD151 on CD3+ cells in women with GG genotype compared to women with **(E)** CC genotype without *Ct* stimulation (green histograms), after *Ct* stimulation (orange histograms) and after anti-CD3/CD28 stimulation (blue histograms). The x-axis indicates the fluorescence intensity of CD151. **(F)** Box and whisker plots summarize the MFIs of CD151 in the different genotype groups with and without *Ct* stimulation. For MFI, intensities were significantly greater in the CC group compared to GG group with and without stimulation by Wilcoxon Rank Sum Test. Wilcoxon signed rank test indicated *Ct* stimulation significantly increased the MFI expression of CD151 in both groups, *:P < 0.05; **:P < 0.01.

We compared T cell CD151 expression in women with CC and GG genotypes after a 7-day culture of PBMCs in media, media with anti-CD3/CD28, or killed *Ct*, and determined that frequencies of CD151+CD3+ lymphocytes were increased in in both unstimulated and stimulated cells of women with the CC genotype (**Figure 6**). Although incubation with *Ct* led to increases in numbers of CD151+ T cells in both genotype groups, the increases were not statistically significant (**Figure 6C**). Nevertheless, the mean fluorescent intensities (MFIs) of CD151 expression per cell concatenated from 6 women with CC genotype was higher than ones from women with GG genotype with and without *Ct* stimulation and increased in both genotype groups after stimulation with *Ct* (**Figures 6D, E**), and all these changes were statistically significant when quantified at the individual subject level (**Figure 6F**). Cell expression of CD151 also increased in both genotype groups with CD3/CD28 stimulation, demonstrating the response also occurs with non-specific stimulation of the T cell receptor (**Figures 6D, E**). Frequencies of CD4+ (**Supplementary Figure 1A**), CD8+ (**Supplementary Figure 1B**), naive (**Supplementary Figure 1C**), and memory (**Supplementary Figure 1D**) CD3+ lymphocytes were not different among genotype groups with or without stimulation. However, *Ct* stimulation led to a downward shift in frequencies of naïve CD3+ lymphocytes and a corresponding increase in memory CD3+ lymphocytes in both genotypes (**Supplementary Figures 1C, D**, respectively), indicating *Ct* responsiveness led to memory T cell proliferation in both patient subgroups. Taken together, these data indicate that increased frequencies of CD151+CD3+ lymphocytes in the CC genotype are not due to differences in overall numbers of T cells or alterations in relative frequencies of CD4 or CD8 T cells when compared to the GG genotype (**Figure 6**).

**Figure 7:**
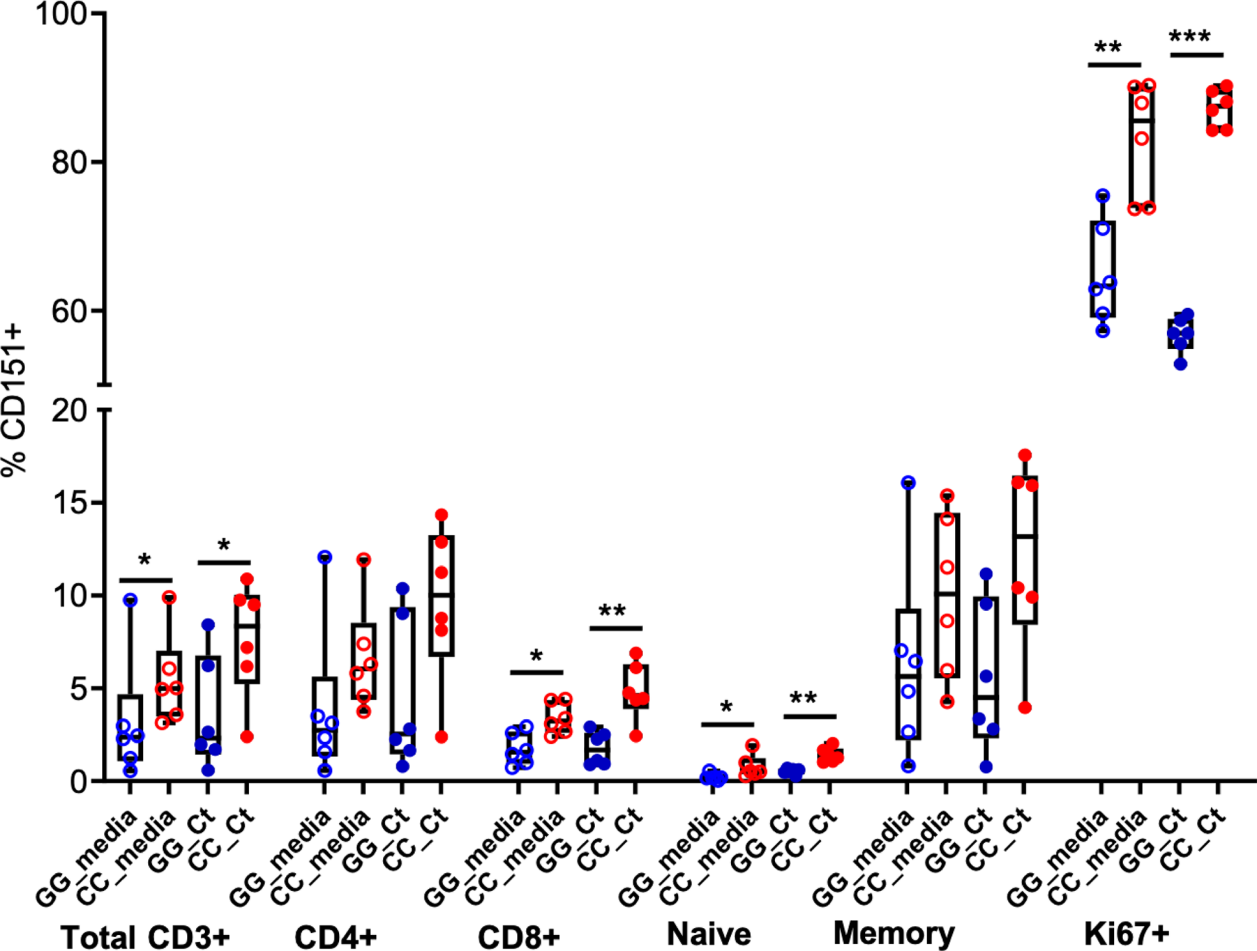
CD151 is enriched in memory and Ki67+ T cells. Box and whisker plots indicate frequencies of CD151+ cells among total CD3+ cells, CD4, CD8, naïve, memory and Ki67+ T cells in GG (n = 6; blue circles) and CC (n = 6; red circles) genotype groups, without (open circles) and with (closed circles) *Ct* stimulation for seven days. The boundaries of the box indicate the 25th and 75th percentiles; the line within the box indicates the median; whiskers indicate the 10th and 90th percentiles. Each dot represents one subject. The relative frequencies of CD151+ lymphocytes were significantly increased in the CC group with and without *Ct* stimulation compared to GG group among total CD3+ cells, CD8, naïve and Ki67+ T cells by Wilcoxon Rank Sum Test. *:P < 0.05; **:P < 0.01; ***:P < 0.005. No statistical differences were detected between media and Ct-stimulated groups for any cell type.

Finally, we characterized the presence of CD151 expression on T cell subpopulations after stimulation (**Figure 7**). As reported above, frequencies of CD151+CD3+ cells were increased in CC genotype women and *Ct* stimulation led to marginal increases in CD151+ cell frequencies. Flow cytometry using T cell specific markers revealed these patterns were consistent across T cell subsets. Frequency of CD151 expression was higher among CD4 T cells compared to CD8 T cells, rare among naïve T cells, but easily detected in memory T cells with the highest expression detected in Ki67+ proliferating T cells. Higher CD151 expression was consistently observed in T cells from women with the CC genotype where endometrial infection was reduced, suggesting that CD151 may play a protective role during *Ct* infection (**Figure 7**).

## Discussion

We performed eQTL mapping and mediation tests to link gene expression-associated loci with presence of endometrial infection and provided evidence for 81 eGenes mediating the cis-eQTL effects on ascending infection (FDR < 0.2), inherently associated with altered disease risk.

Expression of multiple eGenes important for formation of epithelial tight junctions was downregulated in women with endometrial infection. Compromised epithelial integrity may lead to enhanced *Ct* spread directly, and accompanying decreases in expression of molecules engaged in actin cytoskeleton activities, *IQGAP2*, and cell-cell matrix interactions, such as *SGCE*, could result in dysregulated immune cell adhesion and migration, further enhancing spread of infection. The intracellular niche of *Ct* imposes the need and/or opportunity for the bacterium to utilize host cell metabolic pathways for its benefit. Differential expression of eGenes involved in cellular iron acquisition, lipid and fatty acid modification, tryptophan and glucose metabolism, which could directly affect chlamydial fitness, growth, and replication, were associated with altered risk of *Ct* ascension (**Figure 2**). Changes in tryptophan metabolism could also exert indirect effects on *Ct* ascension. Tryptophan catabolites inhibit both T cell and natural killer (NK) cell proliferation and activation (58), immune cell subsets important in *Ct* infection resolution. Alterations in glucose metabolism could also affect *Ct* infection indirectly *via* effects on T cell functionality (82).

eGenes involved in innate immune responses, neutrophil migration and activation, and type I interferon signaling were upregulated in women with endometrial infection, suggesting that activation of these pathways fail to prevent *Ct* ascension (**Figure 2**). Prior work in mice and humans indicate that enhanced TLR activation (83–85), phagocyte activation (71, 86, 87), and type I interferons (3, 69) are associated with increased disease development, rather than protection. Thus, host genetics may play an important role in driving enhanced inflammatory responses that are ineffective for host defense against *Ct* and lead to disease.

Endometrial infection was associated with decreased expression of eGenes involved in generation of adaptive T cell immunity. This correlation agrees with the central role of T cells, particularly *Ct-*specific CD4 T cells, in resolution and protection from *Ct* infection. Women with endometrial infection had lower expression of genes for MHC Class II molecules, HLA-DRB1 and HLA-DPB1. A recent study reported that a variety of HLA-DRB1 alleles/haplotypes were associated with altered *Ct* infection risk, and differential rate of clearance in a cohort of Columbian women (88); HLA-DPB1 alleles were not examined.

The finding of decreased expression of eGene for FcRL3 in women with endometrial infection is particularly interesting given its role in mucosal immunity through binding of secretory IgA. Agarwal et al. (77) demonstrated the importance of pathogen-specific secretory IgA binding of FcRL3 on T cells to inhibit their regulatory function and drive their conversion to proinflammatory Th17-like cells that could combat pathogens breaching the mucosa.

We determined that women with the GG genotype for a lead eSNP for *CD151* exhibited significantly decreased whole blood and T cell CD151 expression compared to women with GC and CC genotypes that associated with presence of endometrial infection. We experimentally confirmed that the cis effect of the lead eSNP of *CD151* on expression of CD151 by T cells was determined by genotype both before and after *in-vitro* stimulation with *Ct* or anti-CD3/CD28, suggesting this cis eQTL can be considered as a candidate biomarker for ascension.

In cooperation with other tetraspanin molecules in tetraspanin-enriched microdomains (TEMs), CD151, regulates cell adhesion and migration through physical and functional interactions with integrins (89). CD151 strongly associates with LFA-1 and the β1 integrin family integrins (75). Complexes form between CD151 and αLβ2 (LFA-1) and between CD151 and α4β1 integrin (VLA-4). LFA-1 has an essential role in mediating firm adhesion and transendothelial migration of T cells from the bloodstream into tissues. T cell expression of CD151 has been shown to be important in T cell trafficking towards chemokine gradients and to inflamed mucosal sites *in vivo*. In addition to their role in T cell migration, CD151-enriched microdomains stabilize the immune synapse at the T cell/antigen presenting cell (APC) interface (76). Consistent with its interactions with integrins, silencing of CD151 expression diminishes relocalization of α4β1 to the IS, resulting in reduced phosphorylation of integrin targets FAK and ERK1/2, and blunting of IL-2 secretion (76). We determined that not only were the frequencies of CD151+ T cells lower in women with the GG genotype, CD151 expression levels per T cell were also reduced, which would further decrease migratory and T cell activation functions augmented by CD151.

Seu et al. compared human CD151+ and CD151- T cells using an *in vitro* kinome array and determined that CD151 actively changes cell cycle control and cell death process motifs, leading to a hyperresponsive proliferation phenotype (13). Consistent with this report, we observed marked enrichment of CD151 in proliferating Ki67+ T cells after a 7 day culture of PBMCs in media alone or media containing inactivated *Ct*, regardless of genotype. However, significant differences in CD151 expression were observed across all T cell subsets according to genotype, and especially among frequencies of Ki67+CD151+ T cells, with significantly higher frequencies of double positive T cells being present in CC genotype women who had a reduced risk of endometrial infection. We also observed CD151 expression frequency was higher in memory T cells versus naïve T cells, as reported by Seu et al. (13). Our study examined PBMCs from *Ct-*infected women after 7 days in culture with media or inactivated *Ct*, and found that CD151 expression was higher in CD4 T cells compared to CD8 T cells, particularly for women with the CC genotype, where *Ct* stimulation led to further increases in CD151 expression (**Figure 6**). Perez et al. (12) and Seu et al. (13) reported CD151 expression frequencies were generally higher on CD8 T cells than CD4 T cells when freshly thawed PBMCs from healthy human donors were analyzed. We determined that frequencies of Ki67+ T cells were 30% and 36% greater for CD4 versus CD8 T cells from women with GG and CC genotypes, respectively on day 7 of culture with inactivated *Ct* (data not shown). The frequent coexpression of Ki67 and CD151 combined with enhanced proliferation of CD4 T cells after *Ct* stimulation likely explains our detection of CD151 enrichment in the CD4 T cell subset.

In summary, literature supports CD151 is important in CD4 T cell migration, immune synapse stabilization, and T cell proliferation. Our data demonstrate *CD151* allelic differences determined by the lead cis eQTL impact whole blood *CD151* expression, and this correlates with risk of endometrial infection. Furthermore, we detected differences in CD4 and CD8 T cell expression of CD151 that could alter proliferative capability, particularly that of memory T cells where CD151 expression is enriched. Enhanced T cell responses may reduce risk of ascending infection in a host genotype dependent manner. These results are highly consistent with our previous blood transcriptomic network analysis where we determined MHC antigen presentation and T cell signaling pathways were upregulated in women without CT induced endometritis and symptomatic pelvic inflammatory disease (3).

We previously determined candidate infertility loci by conducting GWAS in *Ct-*seropositive women with defined fertility status and investigated their concurrence with SNPs associated with *Ct* ascension in an independent *Ct-*infected cohort with biopsy-diagnosed endometrial infection status (11). This approach enabled functional annotation of SNPs to response pathways that alter risk for ascension. Innate immune response pathways, including type I IFN production, pathways important for T cell activation and function, and tryptophan metabolism were identified, as observed in the current study. A non-overlapping gene set unique to the infertility study included genes specifically related to female reproductive tract health, which were not detected in our current eQTL study of acute *Ct* infection. Unique to the current analysis was a determination of genes involved in immune cell migration. These findings suggest that using *Ct* ascension as an intermediate trait increases the power to identify genetic loci for responses that lead to control of *Ct* infection, which may be underestimated by infertility GWAS.

Another strength of this study is the availability of comprehensive clinical data, multi-omics profiles and biopsy-diagnosed endometrial *Ct* infection in study participants. Furthermore, we conducted eQTL analysis and adopted a statistically rigorous mediation test to link the eQTLs with *Ct* ascension. We identified blood eQTLs associated with ascension, which provide candidate biomarkers for targeted screening and vaccine development. However, identification of genital tract tissue eQTLs is warranted for improving understanding of disease mechanisms.

This study has potential limitations. Endometrial infection was determined by transcervical sampling which may lead to inadvertent contamination of the specimen by microorganisms in the cervix. We minimized contamination by carefully sterilizing the endocervical canal through which a sterile endometrial sampler was placed. Our prior work has demonstrated higher rates of endometrial *Ct and N. gonorrhoeae* among women with acute and subclinical PID (90), determined by histological endometritis, compared to women without PID, lending support to endometrial sampling as a valid tool to detect true upper genital tract infection.

Sample size is also limited due to the difficulty in obtaining endometrial biopsy samples. It has been reported that eGenes whose causal SNPs had small allele frequencies using small sample sizes (e.g. frequency <10% in 100 samples) would have inflated FDR (91). We therefore filtered SNPs with minor allele frequency (MAF) < 10% from further analysis in this study. In addition, our mediation test examined the joint effects of multiple SNPs on ascension rather than effects of individual SNPs. The SNPs are not expected to be eQTLs. eQTL screening was used to prioritize the SNPs and reduce multiple testing. Although the lead eSNP for *CD151* is a very common SNP, it may not necessarily be a causal SNP. The association between this eSNP and ascension could result from a causal variant in linkage disequilibrium with this eSNP. A large independent cohort to validate the findings of this study is warranted and the new method of regulatory element-sequencing may be used to determine functional SNPs (92).

In conclusion, we have successfully identified cis-eQTLs associated with ascending infection. Our study highlights the power of a systematic genetics approach and mediation test in dissecting a complex disease and identifying potential genetic biomarkers and pathways. Recruitment of an independent cohort, replicating the recruitment strategy and sample collection of this cohort is ongoing and will enable reexamination of these ascension associated cis-eQTLs for validation and extend our ability to further investigate *CD151* as a potential genetic biomarker of *Ct* risk.

## Data availability statement

The original contributions presented in the study are included in the article/**Supplementary Material**. Further inquiries can be directed to the corresponding authors.

## Ethics statement

The studies involving human participants were reviewed and approved by The Institutional Review Boards for Human Subjects Research at the University of North Carolina, and the University of Pittsburgh approved the study. The patients/participants provided their written informed consent to participate in this study.

## Author contributions

Conceived and designed the study: XZ and TD. Analyzed the data: WZ, XZ, AK, YL, YW, and YTL. Performed the experiments: AK. Wrote the paper: XZ, TD, WZ and AK. Data collection and interpretation: CO’C, TP, KY, HW, and SH. All authors reviewed the results and the manuscript and approved the final version of the manuscript.

## Funding

This work was supported by the National Institute of Allergy and Infectious Diseases at the National Institutes of Health through R01 AI119164 and U19 AI084024 to TD, and U19 AI144181 to TD, CO’C, and XZ.

## Acknowledgments

The authors thank the women who agreed to participate in this study; Ingrid Macio, Melinda Petrina, Carol Priest, Abi Jett, and Lorna Rabe for their efforts in the clinic and the microbiology laboratory; the staff at the Allegheny County Health Department Sexually Transmitted Disease Clinic for their support.

## Conflict of interest

The authors declare that the research was conducted in the absence of any commercial or financial relationships that could be construed as a potential conflict of interest.

## Publisher’s note

All claims expressed in this article are solely those of the authors and do not necessarily represent those of their affiliated organizations, or those of the publisher, the editors and the reviewers. Any product that may be evaluated in this article, or claim that may be made by its manufacturer, is not guaranteed or endorsed by the publisher.
