# Supplementary figures and images for: Genetic susceptibility loci for Chlamydia trachomatis endometrial infection influence expression of genes involved in T cell function, tryptophan metabolism and epithelial integrity
Source: Front Immunol. 2022 Sep 29;13:1001255. doi: 10.3389/fimmu.2022.1001255 (PMC9562917; doi:10.3389/fimmu.2022.1001255)

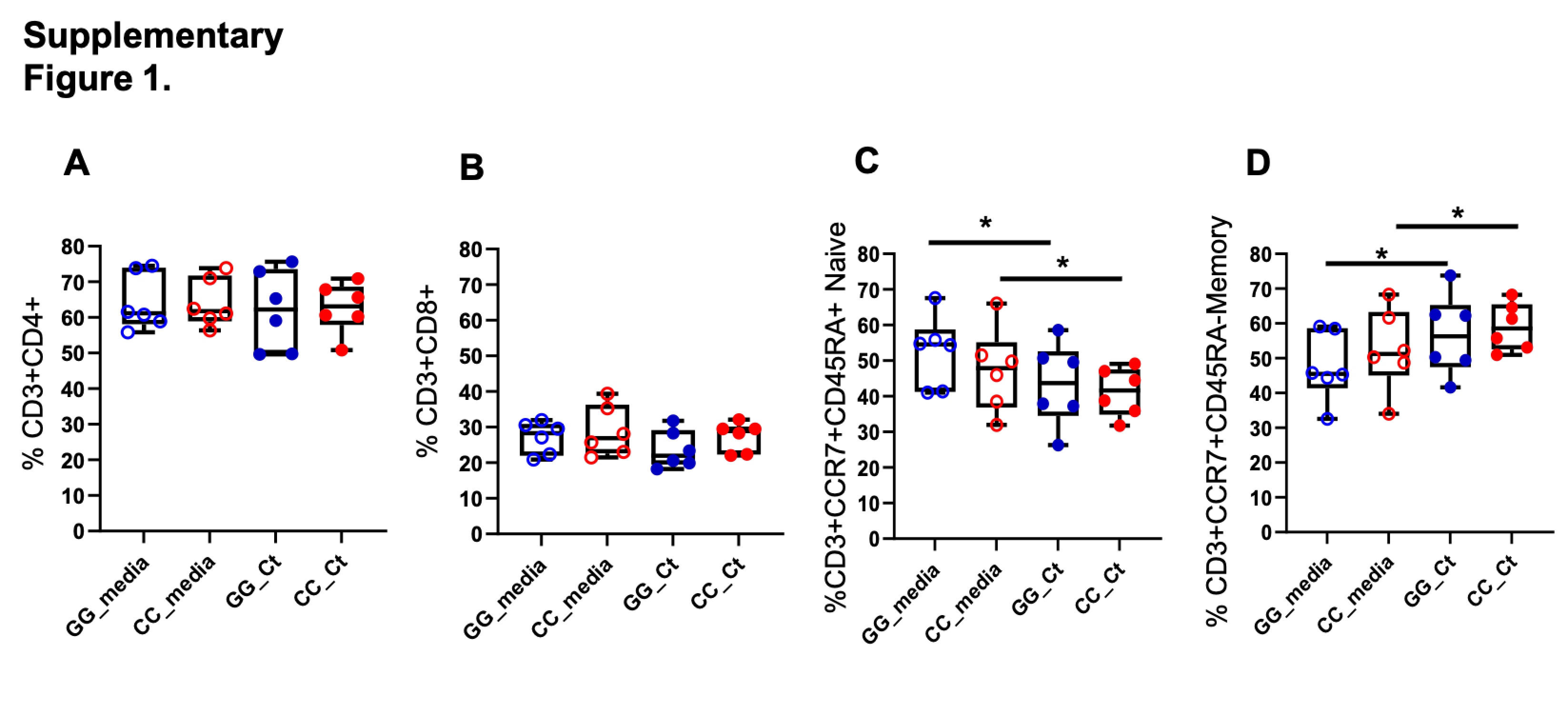

Supplement: Supplementary file 1 [file Image_1.tif]
